# The Treatment of Giant Cell Arteritis in Different Clinical Settings

**DOI:** 10.3389/fimmu.2018.03129

**Published:** 2019-01-24

**Authors:** Alexander Pfeil, Peter Oelzner, Peter Hellmann

**Affiliations:** ^1^Department of Internal Medicine III, Jena University Hospital, Friedrich Schiller University Jena, Jena, Germany; ^2^Chugai Pharma Europe Ltd., Frankfurt am Main, Germany

**Keywords:** giant cell arteritis, tocilizumab, glucocorticoids, clinical settings, relapse, comorbidity

## Abstract

This paper aims to raise awareness of the different disease courses, comorbidities, and therapy situations in patients with giant cell arteritis (GCA), which require a differentiated approach and often a deviation from current treatment guidelines. With the approval of tocilizumab (TOC), which specifically binds to both soluble and membrane-bound IL-6 receptor and inhibits IL-6 receptor-mediated signaling, the spectrum of available effective treatment options has been significantly broadened. TOC yields an extensive range of possible applications that go beyond a glucocorticoid-saving effect. In this context, the treatment of GCA is dependent on the disease course as well as the associated comorbidities. The different stages of GCA in association to co-morbidities require a detailed treatment strategy.

## Introduction

Giant cell arteritis (GCA) is an inflammatory disorder of medium- and large-size arteries affecting people older than 50 years. Classically involved vascular sites include the external carotid branches, the ophthalmic, vertebral, distal subclavian, and axillary arteries as well as the aorta. Segmentary inflammation leads to the occlusion of the vessel and to ischemic complications (1). On the immunological level, a complex interaction between the innate and the adaptive immune system as well as stromal and endothelial cells can be observed (2).

## The Pathophysiology of GCA

Histologically, GCA is characterized by an infiltration of the media with lymphocytes, macrophages, and giant cells (2). Inflammation may show a segmental infestation pattern in which inflammatory and non-inflammatory vascular segments are located side by side (3). The genesis of the disease is unknown. An association between infectious diseases (e.g., parvovirus B19, varicella zoster virus) and the occurrence of GCA is discussed (4–6). With regard to genetic causes, inhomogeneous data are available, whereby HLA-DRB1^*^04 is to be evaluated as a genetic risk factor for the manifestation of GCA (7). On the immunological level, there is a complex interaction between the innate and adaptive immune systems as well as stromal cells and endothelial cells (2). A special role is played by the interleukin-12—T-helper cell 1—interferon- γ–axis and the interleukin-6—T-helper cell 17—interleukin-12 or interleukin-21 axis (8). Interleukin 6-triggered T-cell differentiation to T-helper cell 17 releases various cytokines that control local and systemic inflammatory processes (9). The activation of T-helper cells 1 by interleukin 12 leads to increased secretion of interferon γ, which leads to macrophage activation (9).

Currently, GCA pathophysiology can be diagrammed in two axes which explain the clinical symptoms, the systemic inflammatory response and the vascular occlusion (10). The systemic inflammatory response is associated with the innate immune system. Innate immune systems cells (vascular dendritic cells and monocytes) draw proinflammatory cytokines like Interleukin (IL) 6 which are associated with the production of acute phase proteins in the liver (mainly C-reactive protein) (11, 12). The systemic inflammatory response is glucocorticoid and anti-IL-6 sensitive resulting in reduced clinical symptoms in GCA (11). Vascular occlusion is the ischaemic complication based on vascular remodeling. Activated macrophages or injured vascular smooth muscle cells produced growth factors that trigger vascular remodeling and a myofibroblast differentiation of vascular smooth muscle cells. The myofibroblast migrate into the intimal layer and deposit extracellular matrix proteins resulting in intimal hyperplasia and vascular occlusion in GCA (12). These vascular remodeling is not affected by glucocorticoids and anti-IL-6 therapy (12).

Despite improvements in the understanding of the GCA pathogenesis, glucocorticoids (GC) remain the mainstay treatment of this disease. Unfortunately, relapses are common when the GC dose is tapered, leading to prolonged treatment duration and increased incidence of adverse events (13). Methotrexate (MTX), azathioprine, TNF-alpha blockers, and cyclophosphamide have been proposed as GC-sparing agents or second-line therapy but with conflicting results (14, 15).

Interleukin (IL) 6 plays a central role in the pathogenesis of GCA, and IL-6 serum levels correlate with disease activity and the likelihood of recurrence (16).

Tocilizumab (TOC) is a humanized monoclonal antibody that blocks IL-6 signaling by binding to the alpha chain of the human IL-6 receptor (17). The first results with TOC for treating GCA were published as early as 2011 (18). A first randomized phase II trial followed (19), and finally the randomized phase III study (GiACTA) led to the approval of TOC for the treatment of GCA in 2017 (20).

The initial treatment objective of GCA is rapid disease control by reducing the concentrations of serum acute-phase reactants and freedom from symptoms as well as the prevention of ischemic organ damage. Treatment guidelines have been published by the European League Against Rheumatism (EULAR) (21), the British Society for Rheumatology (BSR) (22), and the French Study Group for Large Vessel Vasculitis (GEFA) (23).

## Topic of This Article

Within the scope of GCA treatment, different disease courses, and therapy situations can be observed, which require a differentiated approach. In addition, existing co-morbidities or GC-induced side effects may demand a deviation from therapy recommendations.

The present article will discuss these aspects and present possible treatment options, especially regarding the approval extension of TOC.

TOC does not only hold potential as a GC-sparing therapy but has already been used in numerous GCA therapy situations. While many case reports primarily focus on the use of the IL-6-receptor blocker in patients with refractory disease, GC dependence or intolerance (18), the randomized TOC studies included both newly diagnosed and recurrent patients (19, 20).

In the registration study GiACTA, 119 patients with newly diagnosed GCA and 132 patients with relapsed GCA were enrolled (20). However, the study design allowed a different initial therapy. Thus, two groups of patients could be included: GC (20–60 mg) at baseline or an existing GC therapy with a duration up to six weeks (20). Therefore, it was possible to start with the combination of GC and TOC as conducted in the smaller phase II trial of Villiger et al. (19) as well as a later add-on of TOC to an initial GC monotherapy.

Various therapy settings can be divided into three main categories:

1. Treatment at new-onset
2. GC-tapering
3. Recurrence after therapy break.

**Figure 1:**
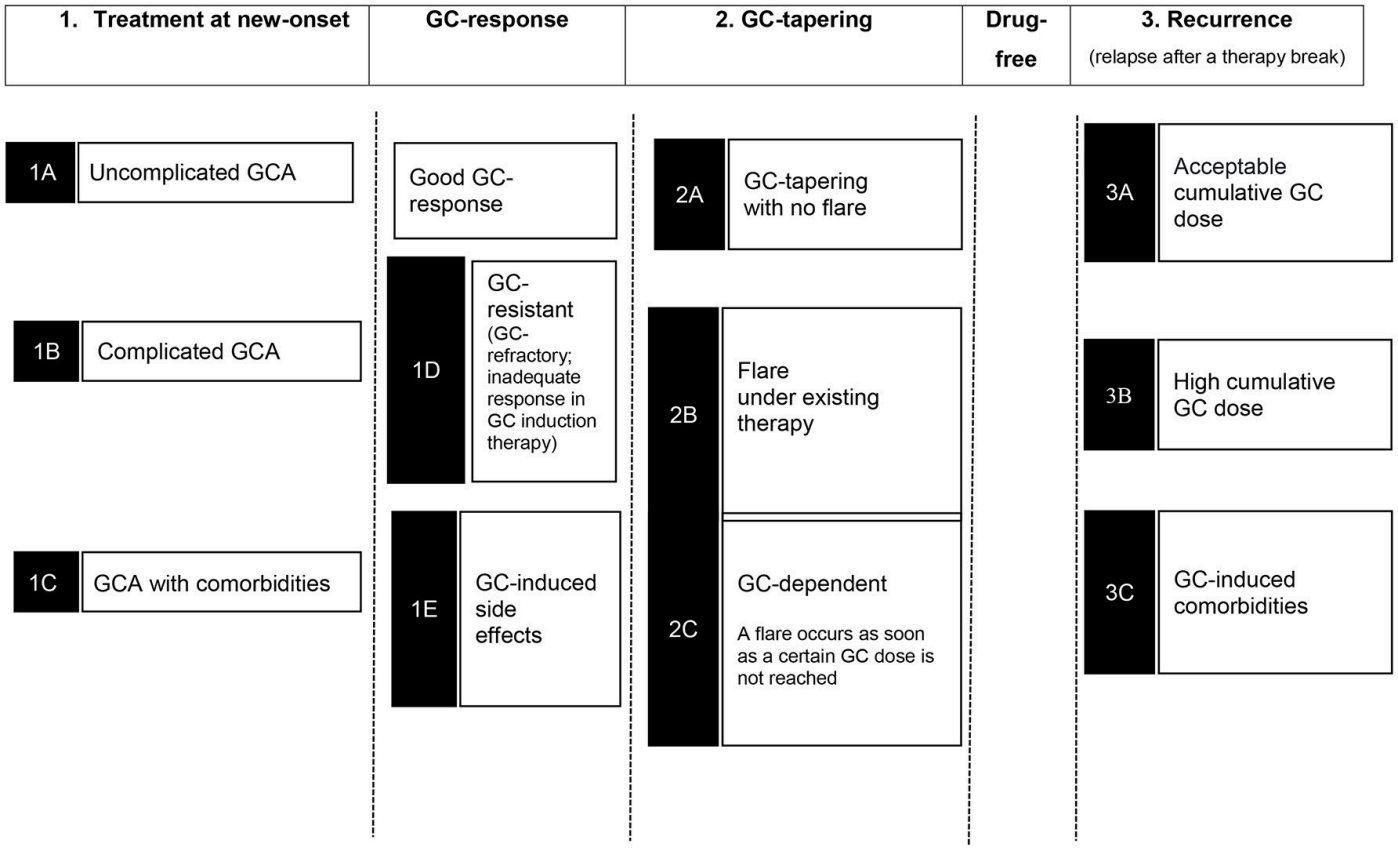
Different clinical settings during GCA treatment.

In addition, further therapy situations such as GC-resistant or refractory disease (lack of response to GC-induction therapy), flare (relapse; a new relapse under therapy), GC-dependency (flare during GC reduction), or patients with co-morbidities and GC-induced side effects can be differentiated (Figure 1).

Table 1 summarizes the substances investigated so far with the patient cohorts and the categorization into therapy situations as shown in Figure 1. Furthermore, the study objectives and the achievement of a GC-free therapy at the end of the trials are shown.

**Table 1:** Investigated drugs in GCA-patients.

**Study drug**	**Trial**	**Total sample size**	**Trial duration (Weeks)**	**Treatment situation corresponding to Figure 1**	**Patient population**	**Trial goal**	**Trial outcome**
Abatacept (24)	RCT	41	52	1A / 3A	New-onset or relapsing GCA	Relapse free survival rate	Positive
Adalimumab (25)	RCT	70	26	1A	New-onset GCA	Portion of patients in Remission	Negative
Anakinra (26)	CR	3	–	1D	Refractory GCA	GC-Sparing	Positive in 2 of 3
Azathioprine (27)	RCT	31	52	2B	GCA or PMR or both GC-dependent	GC-Sparing	Positive only at wk52
Cyclophosphamid (28)	ReS	35	>52	1D	Refractory GCA to GC and MTX or AZA	Remission-induction	Positive
Cyclosporin A (29)	RCT	60	52	1A	New-onset GCA	GC-sparing	Negative
Etanercept (30)	RCT^*^	17	52	1E	GCA with GC induced side effects	Remission at 52 weeks without GC	Positive
Infliximab (15)	RCT	44	22	1A	New-onset GCA	Patients with GC induced remission remained relapse free	Negative, early stop
IV GC Pulse (31)	RCT	27	78	1A	New-onset GCA	GC-sparing	Positive
Leflunomide (32)	ReS	23	51	2B	Persistent or relapsed GCA or PMR	GC-sparing	Positive
Leflunomide (33)	CR	23	NR	2B	GCA and/or PMR with difficulty to tapering GC	Remission induction and GC-sparing	Positive
MTX (34)	RCT^*^	42	96	1A	New-onset GCA	Number of relapses and GC-sparing	Positive
MTX (35)	RCT	21	NR	1A	New-onset GCA	Number of relapses and GC-sparing ^**^	Negative
MTX (15)	RCT^*^	98	52	1A	New-onset GCA	Number of relapses and GC-sparing	Negative
TOC (19)	RCT^*^	30	52	1A/3A	New-onset or Relapsing GCA	Remission at week 12 and GC-sparing	Positive
TOC (20)	RCT^*^	251	52	1A/2B/2C3A	New onset or Relapsing GCA	Sustained GC-free remission^**^	Positive
TOC (36)	ReS	22	16-180	1D/1E	Refractory GCA or GCx side effects	Remission and GC-sparing	Positive
Ustekinumab (37)	PrS	14	52	1D	Refractory GCA	GC-reduction	Positive

RCT, Randomized controlled trials; CR, Case Report; ReS, Retrospective study; PrS, Prospective (registry) study; AZA, Azathiorprin, TOC, Tocilizumab; MTX, Methotrexat NR, Not reported;

* At the end of the study 0 mg GC was reached in the combination arm (e.g., GC + MTX);

** *used SF-36*.

GCA is not a uniform disease. The latest GEFA guidelines differentiate two patient groups at the time of initial diagnosis: (23).

Uncomplicated GCA without ophthalmic involvement and without arteritis of the aorta or its branches

GCA with ophthalmic involvement and GCA with aortoarteritis (non-complicated and asymptomatic involvement of the aorta or its branches)

Accordingly, the GC dosage is adjusted to the risk profile; GCA patients with aortitis require a longer GC therapy due to increased flare rates (38).

## Treatment of New-onset CGA

### Uncomplicated GCA

Although comparative studies on the most effective GC dosage are lacking, the guidelines recommend a GC dosage of 0.7 mg/kg body weight to 1 mg/kg body weight (maximum 60 mg/day) for this patient group (21, 39). Within 24 to 48 h, GC therapy usually leads to a complete resolvement of acute GCA symptoms (40). Regarding the duration of first-line therapy, periods of two to four weeks are discussed (21, 23).

Fifty percent of patients show a good response to GC therapy (41). Both the EULAR (21) and the BSR guidelines (22) recommend the use of GC-sparing immunosuppressants such as MTX to be considered early in therapy planning.

In the GiACTA study, 14% of patients with a GC taper over 26 weeks and 18% of patients with a GC taper over 52 weeks achieved a sustained remission of week 12 to week 52 (20). An increase in the GC dose due to a flare was not necessary.

### Complicated GCA

High, fast-acting intravenous GC pulse therapy (250–1,000 mg over 3 days) is indicated especially in cases of imminent vision loss (22, 23). GC pulse therapy is followed by oral administration of prednisone 1 mg/kg/day (maximum 60 mg/day) (22, 23).

However, in some cases permanent blindness cannot be avoided, because it takes up to five days after the start of a high-dose GC therapy to control the inflammation of the Aa. ciliares posteriores (39).

Furthermore, a recent published study presented on a small study cohort the usefulness of TOC in the treatment of visual symptoms in GCA. The abstract clearly highlighted the non-effectiveness of TOC in blinded patients (42). In this context additionally, comparative studies are required to objectify this point, whereas permanent vision loss occur with an incidence up to 15% (43).

Patients with aortitis often require GC therapy with a duration of over two years and have higher flare rates, resulting in higher cumulative GC dosages (38). Therefore, GC-sparing therapy seems to be useful for this patient group. Interestingly, the subgroup evaluation of the GiACTA study showed that these patients benefited particularly from a weekly dose of TOC (18). Initial studies with a follow-up via positron emission tomography/computed tomography (PET/CT) scanning in a patient with aortitis showed a reduction in the uptake of ^18^F-fluorodeoxyglucose under TOC therapy (44).

### GCA With Comorbidities

None of the guidelines cited discuss a therapeutic approach in patients with a relative contraindication to GC (e.g., severe osteoporosis, diabetes mellitus type 2 difficult to control).

In patients showing GC contraindications at the time of diagnosis, an initial combination of GC and an immunosuppressive agent should be considered. For these patients etanercept and TOC was examined (Table 1). In the GiACTA study, a significant proportion of the patients included had co-morbidities, which significantly restricted the long-term use of GC in particular (45). Patients with relapses had even higher incidences of typical GC-induced (long-term) side effects such as osteoporosis or arterial hypertension. However, a subgroup analysis on the effectiveness and safety of TOC is not yet available.

### GC-Resistant GCA (GC-Refractory)

First-line therapy with GC does not always lead to disease control (which is not defined in GCA). Numerous articles have been published concerning GC-refractory disease (46), and some substances such as cyclophosphamide, anakinra, leflunomide, ustekinumab, and TOC have been investigated under study conditions (Table 1). A rationale for the use of TOC is provided by a current work showing that GC and TOC differ significantly in their effect on regulatory T cells (T reg cells) (47). Consequently, the immunosuppressive effects of GC and TOC are not congruent and the use of TOC in GC-refractory patients is a promising option. Seventeen percent of patients enrolled in the GiACTA study showed refractory disease (45).

### GC-Induced Side Effects

If intolerance or GC-typical side effects occur during GC therapy, the question of GC-sparing therapy arises. In particular, substances and studies that had the goal of completely renouncing GC after a certain treatment period should be taken into account. Publications that included patients in this therapy situation were found for TOC (36) and etanercept (30) (Table 1). MTX is a possible option, but data from a meta-analysis indicate that the onset of action was observed not before 24–36 weeks (14).

## Glucocorticoid-tapering Phase

### GC-Tapering With No Flare

After an initial high-dose GC therapy, GC is slowly reduced (GC tapering), depending on the response. Once again, a uniformly accepted and in clinical studies preferred tapering schedule is not existing. Ideally, a planned GC tapering succeeds as intended and there is no flare-up of the disease. These patients also have the best chance of achieving a very low daily GC dose (< 5 mg) or finishing GC completely after 1 year. In the GiACTA study, 49% of patients with GC monotherapy had a flare within 52 weeks (20). These results support the already published data on GC-monotherapy over 52 weeks (13, 48). In general, a significant number of patients requires permanent GC therapy, and the disease becomes GC-dependent (13).

### Flare (Relapse) During GC-Tapering

A flare (relapse) under existing therapy should be distinguished from a recurrence (flare in the therapy free interval) (48). In some studies and publications, relapse and recurrence are not properly differentiated (23).

A flare during GC-tapering in the GiACTA study is an important criterion as in this case the GC dose could be increased at the physician's discretion. The GC monotherapy arms showed higher flare rates (GC-tapering over 26 weeks 68%; GC-tapering over 52 weeks: 49%) than the TOC study arms including a GC taper over 26 weeks (TOC q1w: 23%; TOC q2w: 26%). Consequently, patients under GC monotherapy had almost twice the cumulative GC doses than patients under combination therapy with TOC (20). Of special importance is also the disease control by a TOC monotherapy starting from the 26th study week (end of the GC taper), exhibiting a continuously low flare rate.

### GC-Dependent

In 40.8% of patients, a certain GC dose cannot be undercut without a new increase in disease activity (46). Especially in this group of patients the question of an additive or intensified immunosuppressive therapy arises. Numerous studies have investigated whether an additional immunosuppressive therapy allows GC-sparing. However, a distinction should be made between studies involving patients in the GC-tapering phase (GC-dependent or side effects) and those initially starting with a combination therapy (Table 1).

The current guidelines of EULAR (21), BSR (22), and GEFA (23) recommend MTX as a GC-sparing agent. It should be noted, however, that the available MTX studies did not include patients with GC-tapering issues but only newly diagnosed patients. In addition, the delayed onset of action under MTX must be considered (2). Furthermore, all three guidelines were published before the approval of TOC and the publication of the GiACTA results. Based on these findings, additionally studies were required which is comparing the effect of the GC- sparing drugs such as TOC and MTX in GC dependent patients.

## Quality of Life (QoL) and Patient Reported Outcome (PRO)

QoL and PRO should be a central component of RCT in view of the frequent GC-induced side effects and its long-term application in GCA. In fact, only two studies (MTX, TOC) assessed SF-36 (Table 1). MTX did not show any improvement of this parameter vs. GC monotherapy (35). In contrast, the TOC study arm revealed a significant improvement in QoL and a significant reduction in fatigue compared to GC monotherapy in the GiACTA study (20).

Since fatigue is a common side effect of MTX, its general use as GC-sparing therapy might be problematic in GCA patients (49).

## Recurrence

A recurrence after a therapy break occurs in about one third of patients (23).

### Acceptable Cumulative GC Dose (Recurrence With No Problems to Take GC)

Patients with recurrent disease, no contraindications to GC as well as no high cumulative CG-dosages (cut-off value is yet not defined) can again be treated with a GC monotherapy according to the published guidelines (21–23).

### Recurrence With High Cumulative GC Dose

Patients suffering a recurrence after 1 or 2 years of GC therapy usually have a correspondingly high cumulative GC dosage and therefore require GC-sparing therapy. In this context, the EULAR guidelines recommend the same “initial” therapy with GC as for new-onset patients (21). According to the EULAR guidelines, an appropriate GC-sparing therapy should be initiated as well. A similar approach is recommended by the BSR (22). No differentiation is made between flare and recurrence (22). Additional immunosuppressive therapy (e.g., MTX) is also recommended. The GEFA guidelines could not identify any study that primarily included patients with relapsing or recurrent disease (23). Therefore, the data situation is weak, and in particular the administration of further immunosuppressants such as MTX (studies only in new-onset GCA patients) should be considered worthy of discussion (23).

### Recurrence With GC-Induced Comorbidities

In everyday clinical practice, however, patients with recurrent disease are a particular challenge as they can develop typical co-morbidities within the first 2–4 years (50). This is shown in the GiACTA study because this group of patients exhibit a higher body weight and body mass index as well as more frequent depression and osteoporosis. In addition, this patient group was more frequently pre-treated with the combination therapy MTX and GC (17 vs. 2% in new-onset patients) (45).

This subgroup evaluation of the GiACTA study suggests that patients with recurrent disease benefit from therapy intensification, as sustained remissions were more frequently achieved in the two TOC study arms compared to the GC monotherapy arms (TOC-QW: 52.8%; TOC-Q2W: 47.8%; GC monotherapy 26 weeks tapering arm: 7.4%; GC monotherapy 52 weeks tapering arm: 14.3%) (45).

RCTs with this patient population are only available for abatacept, leflunomid, and TOC (20, 24, 33). A GC-sparing effect has been shown for TOC in a RCT and for leflunomide in a retrospective evaluation (Table 1). Further, the follow-up evaluation of the Villiger study (19) presented with a median time of 5 months after the last TOC application an GCA-flare in 55% of the patients (51).

## Disease Monitoring

To monitor GCA under the treatment with TOC, traditional acute phase reactants cannot be used to control the disease activity, whereas GCA patients under the treatment with GC present also normal acute phase reactants at the time of disease flare (52). In this context, new biomarkers were required to evaluate disease activity and disease flare. Van Sleen et al. reported in an initial study the usefulness of serum calprotectin as biomarker for the detection of vascular inflammation (53) and Gonzalez et al. reported Osteopontin as a potential predictor for relapse in GCA (54). The value of the different imaging techniques like magnetic resonance imaging and Fluorine-18-fluorodeoxyglucose (FDG) PET/CT to detect disease activity of the Aorta and its branches, especially under GC-treatment is unclear (54, 55). Consequently, new serological and immunological parameters should be evaluated to verify activity and flare of GCA.

## Safety

The IL-6-receptor inhibitor has been approved for the treatment of rheumatoid arthritis (RA) in Europe in 2009 and its safety profile has been published several times (56). The GiACTA study revealed no new safety signals and no gastrointestinal perforations (20).

## Costs

The TOC-therapy in GCA is significantly increased compared to GC-therapy, where at the TOC-therapy is comparable to other biologic drugs in the treatment of rheumatoid arthritis. With ending of the TOC patent protection in 2020 and the implementation of TOC biosimilars the price of TOC will be marked down.

## Future Perspectives

In the course of GCA treatment, numerous situations arise in the clinical routine in which a deviation from therapy guidelines seems to be necessary. However, treatment options as well as co-morbidities are not sufficiently addressed in the recommendations. The approval extension of TOC results in an extensive range of possible applications that go beyond a GC-saving effect. Future studies are necessary to obtain better validated results in efficacy and safety for these treatment situations. New guidelines should also take into account the specific patient profile with regard to the therapy situation and co-morbidities.

## Author Contributions

AP, PO, and PH performed the literature research. AP and PH wrote the first draft of the manuscript, which was then reviewed and edited by PO.

### Conflict of Interest Statement

PH is an employee of Chugai Pharma Europe LTD, Frankfurt am Main, Germany. The remaining authors declare that the research was conducted in the absence of any commercial or financial relationships that could be construed as a potential conflict of interest.

## Abbreviations

BSR: British Society for Rheumatology
CT: Computed tomography
EULAR: European League Against Rheumatism
GCA: Giant cell arteritis
GC: Glucocorticoids
GEFA: French Study Group for Large Vessel Vasculitis
IL-6: Interleukin 6
PET: Positron emission tomography
MTX: Methotrexat
TOC: Tocilizumab.

## References

[1] Aiello PD Trautmann JC McPhee TJ Kunselman AR Hunder GG. Visual prognosis in giant-cell arteritis. Ophthalmology (1993) 100:550–5. 10.1016/S0161-6420(93)31608-8 8479714

[2] Ciccia F Rizzo A Ferrante A Guggino G Croci S Cavazza A. New insights into the pathogenesis of giant cell arteritis. Autoimmun Rev. (2017) 16:675–83. 10.1016/j.autrev.2017.05.004 28479485

[3] Klein RG Campbell RJ Hunder GG Carney JA. Skip lesions in temporal arteritis. Mayo Clin Proc. (1976) 51:504–10. 950804

[4] Gabriel SE Espy M Erdman DD Bjornsson J Smith TF Hunder GG. The role of parvovirus B19 in the pathogenesis of giant cell arteritis: a preliminary evaluation. Arthritis Rheum. (1999) 42:1255–8. 10.1002/1529-0131(199906)42:6&lt;1255::AID-ANR23&gt;3.0.CO;2-P 10366119

[5] Wagner AD Gérard HC Fresemann T Schmidt WA Gromnica-Ihle E Hudson AP. Detection of *Chlamydia pneumoniae* in giant cell vasculitis and correlationwith the topographic arrangement of tissue-infiltrating dendritic cells. Arthritis Rheumatol. (2000) 43:1543–51. 10.1002/1529-0131(200007)43:7<1543::AID-ANR19>3.0.CO;2-8 10902759

[6] Nagel MA White T Khmeleva N Rempel A Boyer PJ Bennett JL. Analysis of Varicella-zoster virus in temporal arteries biopsy positive and negative for giant cell arteritis. JAMA Neurol. (2015) 72:1281–7. 10.1001/jamaneurol.2015.2101 26349037 PMC5110206

[7] Carmona FD Mackie SL Martín JE Taylor JC Vaglio A Eyre S. A large-scale genetic analysis reveals a strong contribution of the HLA class II region to giant cell arteritis susceptibility. Am J Hum Genet. (2015) 96:565–80. 10.1016/j.ajhg.2015.02.009 25817017 PMC4385191

[8] Weyand CM Goronzy JJ. Clinical practice. Giant-cell arteritis and polymyalgia rheumatica. N Engl J Med. (2014) 371:50–7. 10.1056/NEJMcp1214825 24988557 PMC4277693

[9] Ninan J Lester S Hill C. Giant cell arteritis. Best Pract Res Clin Rheumatol. (2016) 30:169–88 10.1016/j.berh.2016.05.001 27421223

[10] Watanabe R Goronzy JJ Berry G Liao YJ Weyand CM. Giant cell arteritis: From pathogenesis to therapeutic management. Curr Treatm Opt Rheumatol. (2016) 2:126–37. 10.1007/s40674-016-0043-x 27298757 PMC4902281

[11] Sammel AM Fraser CL. Update on giant cell arteritis. Curr Opin Ophthalmol. (2018) 29:520–7. 10.1097/ICU.0000000000000528 30138144

[12] Terrades-Garcia N Cid MC. Pathogenesis of giant-cell arteritis: how targeted therapies are influencing our understanding of the mechanisms involved. Rheumatology (2018) 57(Suppl. 2):ii51–62. 10.1093/rheumatology/kex423 29982777

[13] Proven A Gabriel SE Orces C O'Fallon WM Hunder GG. Glucocorticoid therapy in giant cell arteritis: duration and adverse outcomes. Arthritis Rheum. (2003) 49:703–8. 10.1002/art.11388 14558057

[14] Mahr AD Jover JA Spiera RF Hernández-García C Fernández-Gutiérrez B Lavalley MP. Adjunctive methotrexate for treatment of giant cell arteritis: an individual patient data meta-analysis. Arthritis Rheum. (2007) 56:2789–97. 10.1002/art.22754 17665429

[15] Hoffman GS Cid MC Rendt-Zagar KE Merkel PA Weyand CM Stone JH. Infliximab for maintenance of glucocorticosteroid-induced remission of giant cell arteritis: a randomized trial. Ann Intern Med. (2007) 146:621–30. 10.7326/0003-4819-146-9-200705010-00004 17470830

[16] Hernández-Rodríguez J Segarra M Vilardell C Sánchez M García-Martínez A Esteban MJ. Tissue production of pro-inflammatory cytokines (IL- 1beta, TNFalpha and IL-6) correlates with the intensity of the systemic inflammatory response and with corticosteroid requirements in giant-cell arteritis. Rheumatology (2004) 43:294–301. 10.1093/rheumatology/keh058 14679293

[17] Mihara M Kasutani K Okazaki M Nakamura A Kawai S Sugimoto M Tocilizumab inhibits signal transduction mediated by both mIL-6R and sIL-6R, but not by the receptors of other members of IL-6 cytokine family. Int Immunopharmacol. (2005) 5:1731–40. 10.1016/j.intimp.2005.05.010 16102523

[18] Leuchten N Aringer M. Tocilizumab in the treatment of giant cell arteritis. Immunotherapy (2018) 10:465–72. 10.2217/imt-2017-0182 29504436

[19] Villiger PM Adler S Kuchen S Wermelinger F Dan D Fiege V Tocilizumab for induction and maintenance of remission in giant cell arteritis: a Phase II, randomized, double-blind, placebo-controlled trial. Lancet (2016) 387:1921–7. 10.1016/S0140-6736(16)00560-2 26952547

[20] Stone JH Tuckwell K Dimonaco S Klearman M Aringer M Blockmans D Trial of tocilizumab in giant-cell arteritis. N Engl J Med. (2017) 377:317–28. 10.1056/NEJMoa1613849 28745999

[21] Mukhtyar C Guillevin L Cid MC Dasgupta B de Groot K Gross W. EULAR recommendations for the management of large vessel vasculitis. Ann Rheum Dis. (2009) 68:318–23. 10.1136/ard.2008.088351 18413441

[22] Dasgupta B Borg FA Hassan N Alexander L Barraclough K Bourke B. BSR and BHPR guidelines for the management of giant cell arteritis. Rheumatology (2010) 49:1594–7. 10.1093/rheumatology/keq039a 20371504

[23] Bienvenu B Ly KH Lambert M Agard C André M Benhamou Y.. Management of giant cell arteritis: recommendations of the French study Group for Large Vessel Vasculitis (GEFA). Rev Med Interne. (2016) 37:154–65. 10.1016/j.revmed.2015.12.015 26833145

[24] Langford CA Cuthbertson D Ytterberg SR Khalidi N Monach PA Carette S Vasculitis clinical research consortium. a randomized, double-blind trial of abatacept (CTLA-4Ig) for the treatment of giant cell arteritis. Arthritis Rheum. (2017) 69:837–45. 10.1002/art.40044 PMC5378642 28133925

[25] Seror R Baron G Hachulla E Larroche C Puéchal X Maurier F Adalimumab for steroid sparing in patients with giant cell arteritis: results of a multicentre randomized controlled trial. Ann Rheum Dis. (2014) 73:2074–81. 10.1136/annrheumdis-2013-203586 23897775

[26] Ly KH Stirnemann J Liozon E Michel M Fain O Fauchais AL. Interleukin-1 blockade in refractory giant cell arteritis. Joint Bone Spine (2014) 81:76–8. 10.1016/j.jbspin.2013.06.004 23890680

[27] De Silva M Hazleman BL. Azathioprine in giant cell arteritis/polymyalgia rheumatica: a double-blind study. Ann Rheum Dis. (1986) 45:136–8 10.1136/ard.45.2.136 3511861 PMC1001834

[28] Loock J Henes J Kötter I Witte T Lamprecht P Schirmer M. Treatment of refractory giant cell arteritis with cyclophosphamide:a retrospective analysis of 35 patients from three centres. Clin Exp Rheumatol. (2012) 30(1 Suppl 70):S70–76. 22640650

[29] Schaufelberger C Möllby H Uddhammar A Bratt J Nordborg E. Schaufelberger C Möllby H No additional steroid-sparing effect of cyclosporine A in giant cell arteritis. Scand J Rheumatol (2006) 35:327–329. 10.1080/03009740500474537 16882602

[30] Martínez-Taboada VM Rodríguez-Valverde V Carreño L López-Longo J Figueroa M Belzunegui J. A double-blind placebo controlled trial of etanercept in patients with giant cell arteritis and corticosteroid side effects. Ann Rheum Dis. (2008) 67:625–630. 10.1136/ard.2007.082115 18086726

[31] Mazlumzadeh M Hunder GG Easley KA Calamia KT Matteson EL Griffing WL.. Treatment of giant cell arteritis using induction therapy with high-dose glucocorticoids: a double-blind, placebo-controlled, randomized prospective clinical trial.ArthritisRheum (2006) 54:3310–3318. 10.1002/art.22163 17009270

[32] Diamantopoulos AP Hetland H Myklebust G Diamantopoulos AP Hetland H Myklebust G Leflunomide as a corticosteroid-sparing agent in giant cell arteritis and polymyalgia rheumatica: a case series. Biomed Res Int. (2013) 2013:120638 10.1155/2013/120638 PMC3784071 24106691

[33] Adizie T Christidis D Dharmapaliah C Borg F Dasgupta B. Efficacy and tolerability of leflunomide in difficult-to-treat polymyalgia rheumatica and giant cell arteritis: a case series. Int J Clin Pract. (2012) 66:906–9. 10.1111/j.1742-1241.2012.02981.x 22897467

[34] Jover JA Hernández-García C Morado IC Vargas E Bañares A Fernández-Gutiérrez B. Combined treatment of giant-cell arteritis with methotrexate and prednisone. a randomized, double-blind, placebo-controlled trial. Ann Intern Med. (2001) 134:106–14. 10.7326/0003-4819-134-2-200101160-00010 11177313

[35] Spiera RF Mitnick HJ Kupersmith M Richmond M Spiera H Peterson MG. A prospective, double-blind, randomized, placebo controlled trial of methotrexate in the treatment of giant cell arteritis (GCA). Clin Exp Rheumatol. (2001) 19:495–501. 11579707

[36] Loricera J Blanco R Castañeda S Humbría A Ortego-Centeno N Narváez J. Tocilizumab in refractory aortitis: study on 16 patients and literature review. Clin. Exp. Rheumato (2014) 32(3 Suppl. 82): S79–89 10.1136/annrheumdis-2014-eular.2984 24854377

[37] Conway R O'Neill L O'Flynn E Gallagher P McCarthy GM Murphy CC. Ustekinumab for the treatment of refractory giant cell arteritis. Ann Rheum Dis. (2016) 75:1578–9. 10.1136/annrheumdis-2016-209351 27143653

[38] Muratore F Kermani TA Crowson CS Koster MJ Matteson EL Salvarani C. Large vessel dilatation in giant cell arteritis: a different subset of disease? Arthritis Care Res. (2017) 70:1406–11. 10.1002/acr.23498 29266882

[39] Hellmich B. Management of polymyalgia rheumatica and large vessel vasculitis. Internist (2016) 57:1069–78. 10.1007/s00108-016-0131-x 27631531

[40] Chatterjee S Flamm SD Tan CD Rodriguez ER. Clinical diagnosis and management of large vessel vasculitis: giant cell arteritis. Curr Cardiol Rep. (2014) 16:498. 10.1007/s11886-014-0498-z 24893935

[41] Restuccia G Boiardi L Cavazza A Catanoso M Macchioni P Muratore F. Long-term remission in biopsy proven giant cell arteritis: a retrospective cohort study. J Autoimmun. (2017) 77:39–44. 10.1016/j.jaut.2016.10.002 27742223

[42] Goercke MC Loricera J Peña DP Morales CD Alvarez DA Narváez JN Utility of Tocilizumab in visual affection of patients with giant cell arteritis. 2018 ACR/ARHP Annual Meeting. Arthritis Rheumatol. (2018) 70(Suppl. 10). Abstract Number: 2755

[43] Borchers AT Gershwin ME. Giant cell arteritis: a review of classification, pathophysiology, geoepidemiology and treatment. Autoimmun Rev. (2012) 11:A544–54. 10.1016/j.autrev.2012.01.003 22285588

[44] Vitiello G Orsi Battaglini C Carli G Radice A Matucci A Vultaggio A. Tocilizumab in giant cell arteritis: a real-life retrospective study. Angiology (2018) 69:763–9. 10.1177/0003319717753223 29343075

[45] Tuckwell K Collinson N Dimonaco S Klearman M Blockmans D Brouwer E. Newly diagnosed vs. relapsing giant cell arteritis: baseline data from the GiACTA trial. Semin Arthritis Rheum. (2017) 46:657–64. 10.1016/j.semarthrit.2016.11.002 27998620

[46] Kötter I Henes J Wagner A Loock J Gross WL. Does glucocorticosteroid-resistant large-vessel vasculitis (giant cell arteritis and Takayasu arteritis) exist and how can remission be achieved? A critical review of the literature. Clin Exp Rheumatol. (2012) 30(1 Suppl. 70): S114–29. 22640655

[47] Miyabe C Miyabe Y Strle K Kim ND Stone JH Luster AD. An expanded population of pathogenic regulatory T cells in giant cell arteritis is abrogated by IL-6 blockade therapy. Ann Rheum Dis. (2017) 76:898–905. 10.1136/annrheumdis-2016-210070 27927642 PMC5744591

[48] Martinez-Lado L Calviño-Díaz C Piñeiro A Dierssen T Vazquez-Rodriguez TR Miranda-Filloy JA. Relapses and recurrences in giant cell arteritis: a population-based study of patients with biopsy-proven disease from northwestern Spain. Medicine (2011) 90:186–93. 10.1097/MD.0b013e31821c4fad 21512412

[49] Summary of product characteristics MTX Available online at: https://www.ema.europa.eu/en/medicines/human/EPAR/nordimet

[50] Wilson JC Sarsour K Collinson N Tuckwell K Musselman D Klearman M. Serious adverse effects associated with glucocorticoid therapy in patients with giant cell arteritis (GCA): a nested case-control analysis. Semin Arthritis Rheum. (2017) 46:819–27. 10.1016/j.semarthrit.2016.11.006 28040244

[51] Adler S Reichenbach S Kuchen S Wermelinger F Dan D Seitz M Villiger PM Termination of Tocilizumab-treatment in giant cell arteritis: follow-up of Patients after the RCT (ClinicalTrials.gov registration number: NCT01450137). Arthritis Rheumatol. (2016) 68(Suppl 10). Available online at: https://acrabstracts.org/abstract/termination-of-tocilizumab-treatment-in-giant-cell-arteritis-follow-up-of-patients-after-the-rct-clinicaltrials-gov-registration-number-nct01450137/

[52] Stone JH Tuckwell K Dimonaco S Klearman M Aringer M Blockmans D Acute phase reactant levels and prednisone doses at disease flare in patients with giant cell arteritis: prospective data from the giacta trial. Ann Rheum Dis. (2018) 77(Suppl.):A1120 10.1136/annrheumdis-2018-eular.2719

[53] van Sleen Y Hoekstra M Bijzet J Abdulahad WH Boots AMH Brouwer E Serum IL-6, SAA and calprotectin as biomarkers in giant cell arteritis and polymyalgia rheumatic. Arthritis Rheumatol. (2017) 69 (Suppl. 10). Available online at: https://acrabstracts.org/abstract/serum-il-6-saa-and-calprotectin-as-biomarkers-in-giant-cell-arteritis-and-polymyalgia-rheumatica/

[54] Hauenstein C Reinhard M Geiger J Markl M Hetzel A Treszl A. Effects of early corticosteroid treatment on magnetic resonance imaging and ultrasonography findings in giant cell arteritis. Rheumatology (2012) 51:1999–20 10.1093/rheumatology/kes153 22772317

[55] Nielsen BD Gormsen LC Hansen IT Keller KK Therkildsen P Hauge EM. Three days of high-dose glucocorticoid treatment attenuates large-vessel 18F-FDG uptake in largevessel giant cell arteritis but with a limited impact on diagnostic accuracy. Eur J Nucl Med Mol Imaging (2018) 45:1119–28. 10.1007/s00259-018-4021-4 29671039

[56] Bannwarth B Richez C. Clinical safety of tocilizumab in rheumatoid arthritis. Expert Opin Drug Saf. (2011)10:123–3. 10.1517/14740338.2011.537256 21121872

